# Long non-coding RNAs could act as vectors for paternal heredity of high fat diet-induced obesity

**DOI:** 10.18632/oncotarget.18138

**Published:** 2017-05-24

**Authors:** Tian An, Teng Zhang, Fei Teng, Jia-Cheng Zuo, Yan-Yun Pan, Yu-Fei Liu, Jia-Nan Miao, Yu-Jie Gu, Na Yu, Dan-Dan Zhao, Fang-Fang Mo, Si-Hua Gao, Guangjian Jiang

**Affiliations:** ^1^ Diabetes Research Center, Beijing University of Chinese Medicine, Beijing, China; ^2^ State Key Laboratory of Stem Cell and Reproductive Biology, Institute of Zoology, Chinese Academy of Sciences, Beijing, China; ^3^ The Third Affiliated Hospital, Beijing University of Chinese Medicine, Beijing, China

**Keywords:** long non-coding RNA, paternal inheritance, genetic vector, obesity

## Abstract

Long non-coding RNAs (lncRNAs) play an important role in epigenetic regulation, and abnormalities may lead to male infertility. To investigate whether lncRNAs are involved in intergenerational inheritance of obesity and obesity-induced decline in fertility, we divided mice into obesity (F0 mice fed a high-fat diet, F0-HFD) and non-obese (F0 mice fed normal chow, F0-NC) model groups and their male offspring (F1-HFD and F1-NC, respectively). We examined the differences in the expression levels of lncRNAs and mRNAs in the F0-HFD/F0-NC and F1-HFD/F1-NC groups. The results revealed similar expression patterns in the F1-HFD/F0-HFD groups at both the lncRNA and mRNA levels. The maximum difference in the lncRNA expression was observed between the F0-HFD and F0-NC groups. The differentially expressed lncRNA targets and mRNAs identified in our study are mainly involved in GnRH signalling pathway, metabolic process, and Hippo signalling pathway; similarly expressed lncRNAs and mRNAs in F1-HFD/F0-HFD are closely linked with G-protein coupled receptor signalling pathway, pancreatic polypeptide receptor activity, and lysine biosynthesis, which may play an important role in the molecular mechanism of intergenerational inheritance of obesity. Furthermore, potential genes that might play important roles in the pathogenesis of obesity-related low fertility were revealed by lncRNA-and mRNA-interaction studies based on the microarray expression profiles. In conclusion, we found that lncRNA could be involved in obesity-induced infertility by expressing abnormalities, which could act as genetic vectors of paternal inheritance of obesity.

## INTRODUCTION

Over the past 30 years, obesity in males in the reproductive age has tripled, concomitant with the increase in male infertility [1]. Obesity can have a negative impact on male reproductive potential by reducing sperm density and motility [2, 3] and increasing the release of reactive oxygen species (ROS), which can cause DNA damage and influence plasma membrane integrity in sperm [4, 5]. Male offspring from high fat diet (HFD) fathers have a high sensitivity to HFD-induced metabolic and reproductive disturbances [6]. Genetic and epigenetic changes in the spermatozoa may explain paternal programming of offspring phenotypes induced by paternal obesity [7]. Inter/transgenerational phenotype transmission is associated with histone modifications, altered DNA methylation, and noncoding RNA transcripts [8–10]. DNA methylation is required for spermatogenesis, and it has been demonstrated that diet-induced paternal obesity regulates germ cell methylation status and causes metabolic disorders in two generations of mice [11, 12]. Paternal obesity changes the expression of insulin-like growth factor 2 (IGF2) in infants by influencing normal IGF2 methylation in spermatozoa [13]. The contribution of the patrilineal phenotype to fertility is not just at the DNA level [14]; sperm RNA can also reflect the quality of spermatogenesis, and its fertilizing capacity and post-fertilisation functions [15].

Long non-coding RNAs (lncRNAs) play an important role in mature sperm. LncRNAs can play a critical role in genomic imprinting [16], adipogenesis and metabolism [17], adipocyte differentiation and development [18]. Interestingly, lncRNA may also play an important role in the context of the expanding sperm RNA-protein network [19, 20]. Our previous study revealed that 7721 lncRNAs are differentially expressed between normal and diabetic sperm groups, suggesting that lncRNA may be a potential regulatory target associated with spermatogenesis in men with diabetes [21]. Cadmium (Cd) exposure can lead to abnormal changes in the expression profile of sperm lncRNAs [22]. Another study found that obesity might be associated with the expression of disorderslncRNA by constructing a circulating lncRNA expression profile in obese and non-obese humans [23].

Although the role of lncRNA in obesity and reproduction has been investigated previously, few studies have examined the role of lncRNA in the context of obesity-induced infertility, and in particular, its influence on offspring spermatogenesis. Therefore, we investigated lncRNAs that are differentially expressed in the spermatozoa of obese and non-obese mice and their offspring by microarray analysis, in order to study the impact of obesity on mice fertility and intergenerational inheritance from lncRNA expression. This study provides a possible lncRNA-related mechanism for obesity-induced male sterility.

## RESULTS

### High fat diet-induced obesity altered the expression of lncRNA and mRNA in mouse sperm

**Figure 1:**
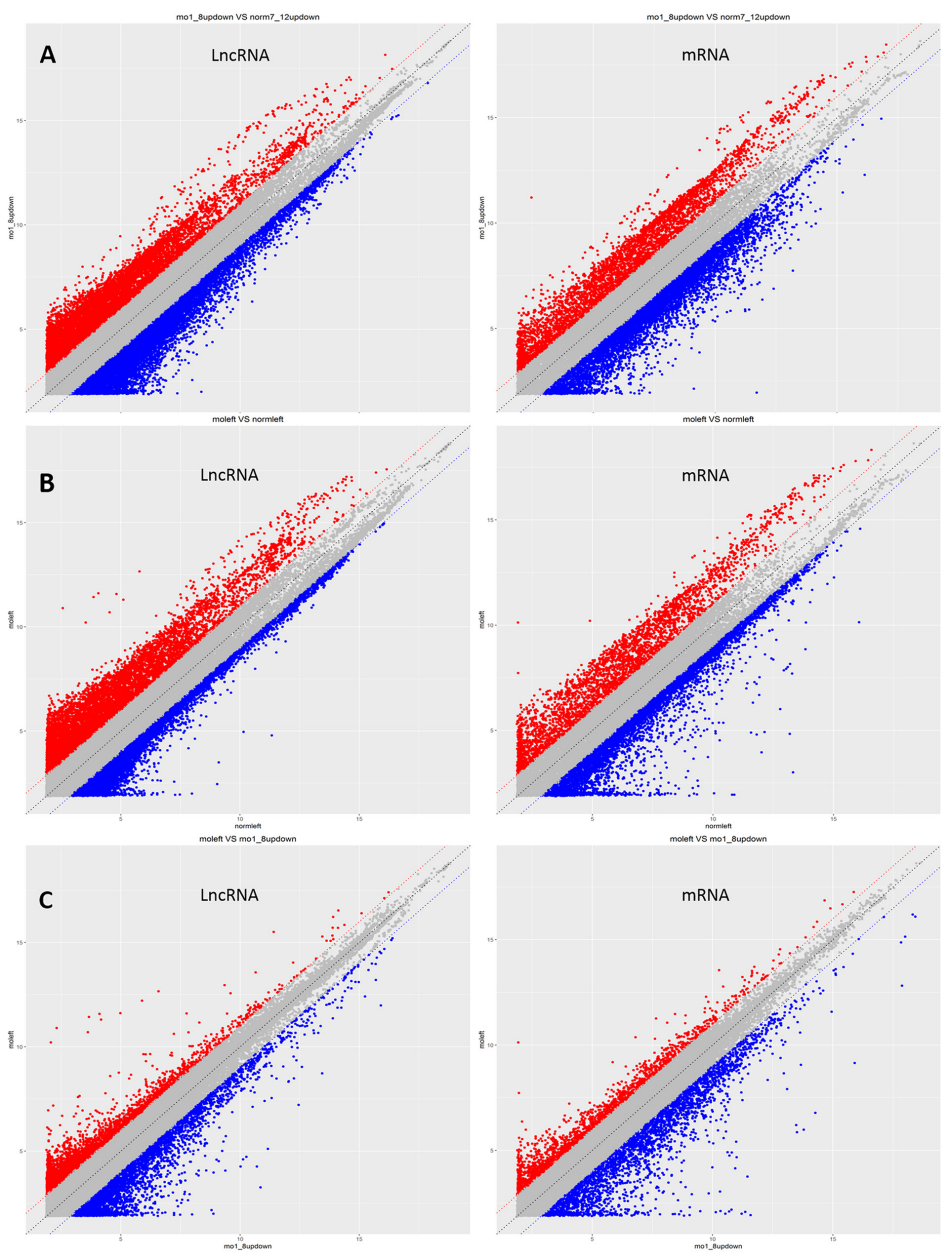
LncRNA scatter plots used to identify differentially expressed lncRNAs in four groups X and Y axes represent averaged normalised signal values of the control and experimental group microarray samples. LncRNAs above the top red line and below the bottom blue line showed greater than 2.0-fold changes in expression between the two compared groups. **(A)** F0-HFD/F0-NC; **(B)** F1-HFD/F1-NC; and **(C)** F1-HFD/F0-HFD.

To identify lncRNAs and mRNAs that are differentially expressed in mice sperm between the obese and normal groups, we carried out microarray analysis of lncRNA and mRNA expression in F0-HFD and F0-NC. In total, 6,354 lncRNAs and 3,370 mRNAs were upregulated while 7,541 lncRNAs and 5,655 mRNAs were downregulated. These results demonstrated that a high-fat diet induced obvious changes in the lncRNA and mRNA expression profiles in the spermatozoa of obese mice. The scatter plot and top 15 differentially expressed lncRNAs and mRNAs between F0-HFD and F0-NC are shown in Figure 1A and Table 1.

**Table 1:** Top 15 differentially expressed lncRNAs and mRNAs in F0-HFD/F0-NC groups

mRNAs		lncRNAs	
Gene_name	Fold change	Gene_name	Fold change
Ngp	428.3283623	NONMMUG009536	24.29140951
Mcam	20.25591007	NONMMUG029430	24.17902223
Gja5	19.53615843	NONMMUG033851	24.11090336
Rhd	19.44082314	NONMMUG018726	24.02573231
Fgg	19.42386373	ENSMUSG00000084785	22.89749706
Yae1d1	18.88614046	Mir32	22.30121644
Gm5148	18.83236784	ENSMUSG00000090026	22.15050983
Rpl6	18.38511123	NONMMUG011486	22.1074696
9530003J23Rik	18.33321626	NONMMUG024207	21.4604214
Fgf15	17.88020039	NONMMUG035045	21.3527503
Agl	17.79835497	NONMMUG037260	21.15388882
3110035E14Rik	17.22059397	NONMMUG031523	20.44854092
Yipf2	17.13240759	NONMMUG003313	20.29794122
Golt1a	17.11676312	Mir206	20.05179104
Gc	17.00442951	ENSMUSG00000097045	19.88438449
B4galt5	0.032368167	NONMMUG016700	0.047472897
Zfp52	0.031937927	NONMMUG017643	0.047422387
Mmp13	0.031746956	NONMMUG017032	0.046587816
Irg1	0.031315495	AI504432	0.045872836
Slc7a11	0.030710923	NONMMUG010104	0.045184993
Adm	0.024873517	NONMMUG016696	0.045036318
Tnfrsf11b	0.024284736	AI504432	0.044699013
Hcar2	0.023393394	NONMMUG016242	0.043215483
Serpinb2	0.022907985	NONMMUG016698	0.042536917
Lif	0.022141803	ENSMUSG00000097418	0.042404113
Hmox1	0.022031532	Mcpt-ps1	0.038230371
Il1a	0.021840103	Gm14023	0.037605847
Il10	0.021072211	NONMMUG005850	0.023176845
Slc7a2	0.007905572	NONMMUG038489	0.022920052
Cxcl3	0.001159635	NONMMUG008794	0.011994736

### The expression patterns of LncRNA and mRNA in male offspring (F1) spermatozoa of obese and normal mice were significantly different

In order to reveal the impact of obesity induced by a high-fat diet on progeny fertility, we examined the expression of lncRNA and mRNA in the spermatozoa of obese and normal mouse offspring. Microarray analysis showed that 5,984 lncRNAs and 2,661 mRNAs were upregulated and 5,543 lncRNAs and 5,846 mRNAs were downregulated in F1-HFD relative to F1-NC mice (Figure 1B). The top 15 significantly differentially expressed lncRNAs and mRNAs in F1-HFD/F1-NC are listed in Table 2.

**Table 2:** Top 15 differentially expressed lncRNAs and mRNAs in F1-HFD/F1-NC groups

mRNAs		lncRNAs	
Gene_name	Fold change	Gene_name	Fold change
Scnn1b	55.989485	NONMMUG003816	187.63459
BC048679	39.563688	E030003E18Rik	117.004727
C8b	18.183759	NONMMUG012756	108.169053
Prss56	17.354256	NONMMUG004111	25.9867966
Hpgds	17.036948	1700023F02Rik	22.7352112
Nutm1	16.77879	1700047L14Rik	21.3072219
Fgf15	16.701433	ENSMUSG00000086534	20.1978776
Rpl6	16.6689	NONMMUG040279	19.4705563
Dnah10	16.504961	NONMMUG008279	18.7480326
Ptprcap	15.671903	NONMMUG016872	18.7032969
Ush1c	15.39363	NONMMUG013706	18.213956
E2f3	14.918401	NONMMUG011486	18.0963333
Agl	14.652562	NONMMUG018726	18.033556
Ssty1	14.198563	ENSMUSG00000090026	17.7970473
Lrrc71	14.059018	ENSMUSG00000099387	17.5358967
Ank1	0.0093276	NONMMUG005810	0.06008047
Sparcl1	0.0091043	ENSMUSG00000084421	0.06005461
Synpo2	0.0083851	NONMMUG023650	0.04962984
Pdlim3	0.0077229	NONMMUG044366	0.0490406
Sh3bgr	0.0076126	NONMMUG023650	0.04353695
Pgm5	0.0071856	ENSMUSG00000085779	0.04183152
Smoc2	0.0067167	ENSMUSG00000097250	0.03925611
Col12a1	0.0067059	NONMMUG034856	0.03851915
Pgm5	0.0059943	NONMMUG025408	0.02739993
Tagln	0.0038105	NONMMUG029313	0.02460214
Cxcl14	0.0037608	ENSMUSG00000097324	0.02170222
Acta1	0.0030556	Mir143hg	0.02041349
Pcp4l1	0.0021702	NONMMUG012048	0.01576866
Pcp4	0.0020343	NONMMUG034976	0.01074036
Myh11	0.0007876	NONMMUG012048	0.0103487

### LncRNA may act as the hereditary vector inducing paternal heredity of obesity (HFD)

To further determine whether intergenerational inheritance of obesity occurs via the expression of sperm lncRNA, we compared lncRNA expression in the spermatozoa and found that 26,531 lncRNAs in F1 and F0 obese mice had the same expression profiles. The opposite of differentially expressed lncRNA is a minority, 1,752 lncRNAs and 1,218 mRNAs were upregulated and 2,160 lncRNAs and 2,382 mRNAs were downregulated in F1-HFD as compared to those in F0-HFD mice (Figure 1C).

### Functional analysis of differentially expressed genes

**Figure 2:**
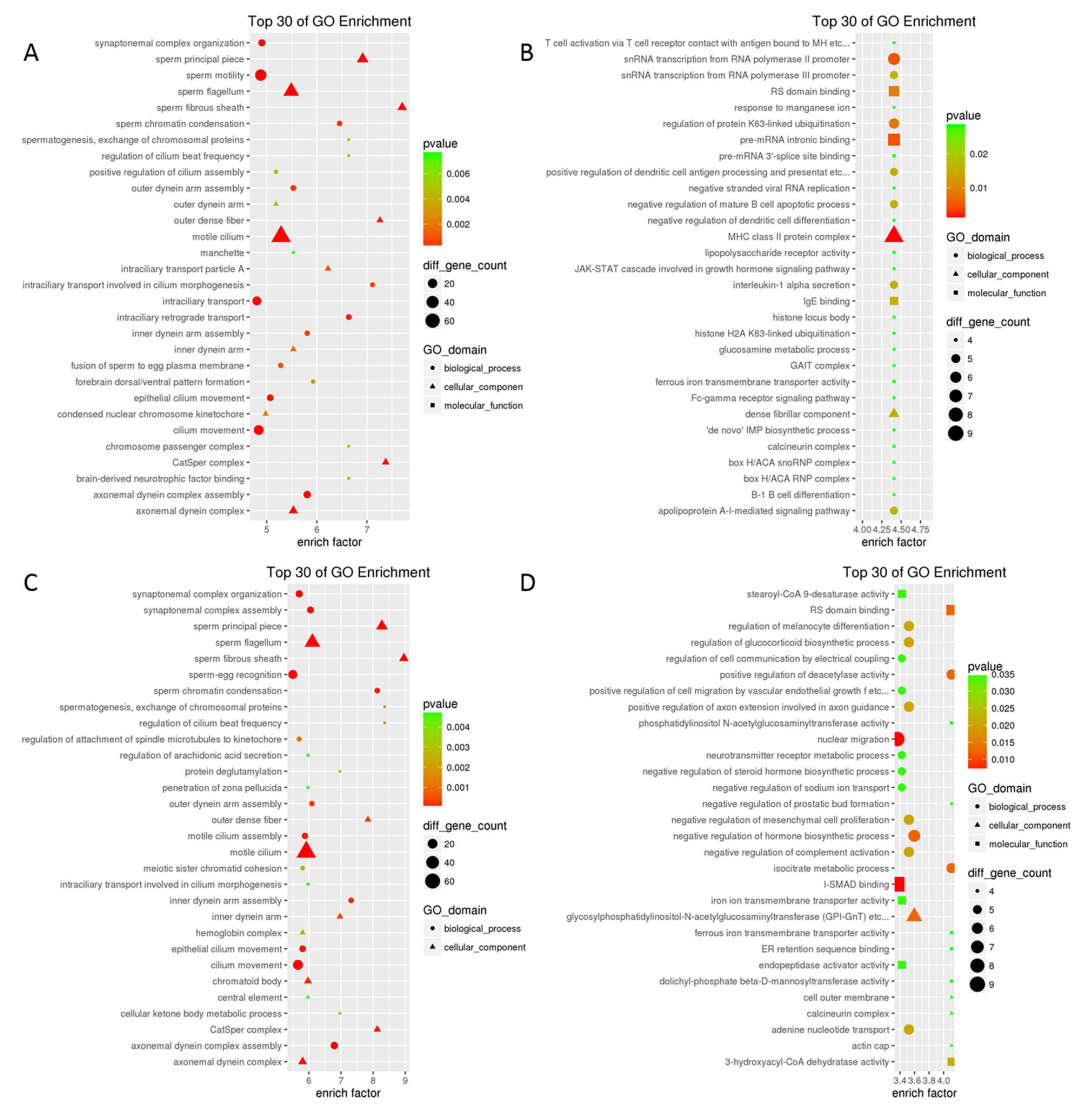
Top 30 enriched GO terms for differentially expressed mRNAs The terms were divided into three categories, including biological process (BP, circles), cellular component (CC, triangles), and molecular function (MF, squares). Size represents the number of enriched genes; colour indicates the degree of enrichment. F0-HFD/F0-NC group **(A)** upregulated; **(B)** downregulated; and F1-HFD/F1-NC group **(C)** upregulated; **(D)** downregulated.

**Figure 3:**
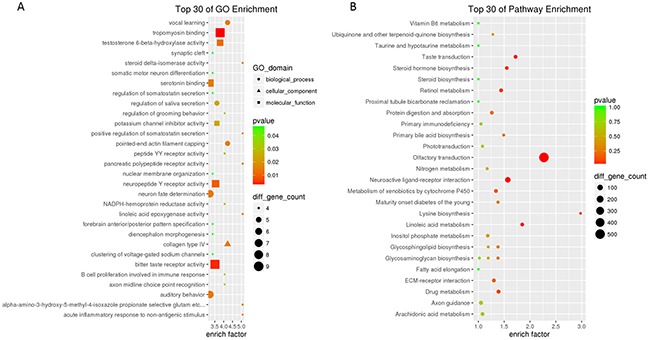
Top 30 enriched GO terms **(A)** and KEGG pathways **(B)** for similarly expressed mRNAs in the F1-HFD and F0-HFD groups.

A Gene Ontology (GO) analysis of differentially expressed mRNAs was carried out to determine their functional significance [24] (Supplementary Table 1). Upregulated transcripts in the F0-HFD/F0-NC and F1-HFD/F1-NC groups were enriched in “spermatogenesis, exchange of chromosomal proteins (biological process)”, “sperm fibrous sheath (cellular component)” while downregulated transcripts were most highly enriched in “MHC class II protein complex” and “positive regulation of deacetylase activity” (Figure 2). The most highly enriched GO terms associated with similarly expressed transcripts in the F1-HFD/F0-HFD groups were “positive regulation of somatostatin secretion”, “steroid delta-isomerase activity” and “collagen type IV” (Figure 3).

### Kyoto Encyclopedia of Genes and Genomes (KEGG) pathway analysis

**Figure 4:**
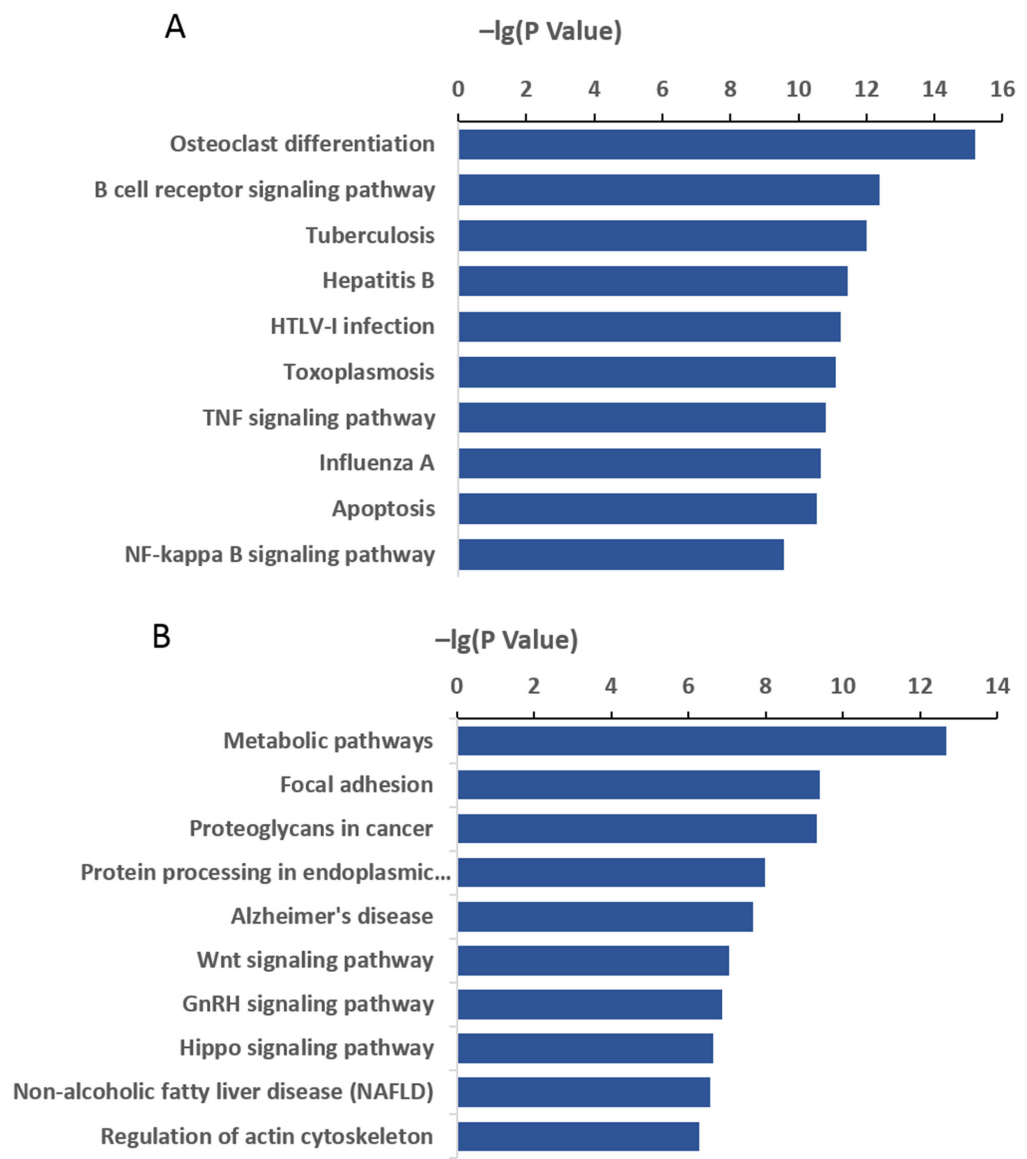
KEGG pathways of differentially expressed mRNAs Vertical and horizontal axes represent the pathway category and enrichment score [−log 10 (P value)] of the pathway, respectively. **(A)** F0-HFD/F0-NC and **(B)** F1-HFD/F1-NC.

Ten pathways were significantly enriched among the differentially expressed transcripts (*P* < 0.05; Supplementary Table 2). The most highly enriched pathway in the F0-HFD/F0-NC groups was “osteoclast differentiation”, and the most enriched pathways in the F1-HFD/F1-NC groups were “metabolic pathways” (Figure 4). Similarly expressed transcripts in the F1-HFD/F0-HFD groups were highly enriched in “G-protein coupled receptor signalling pathway”, “pancreatic polypeptide receptor activity”, and “Lysine biosynthesis” (Figure 3). Many of these pathways were linked to obesity, such as “B cell receptor signalling pathway”, “MAPK signalling pathway”, “GnRH signalling pathway”, “Hippo signalling pathway”, and “fatty acid metabolism”. The number of genes in the top ten pathways significantly enriched among the differentially expressed transcripts are listed in Figure 4.

### Coding-non-coding gene co-expression network

**Figure 5:**
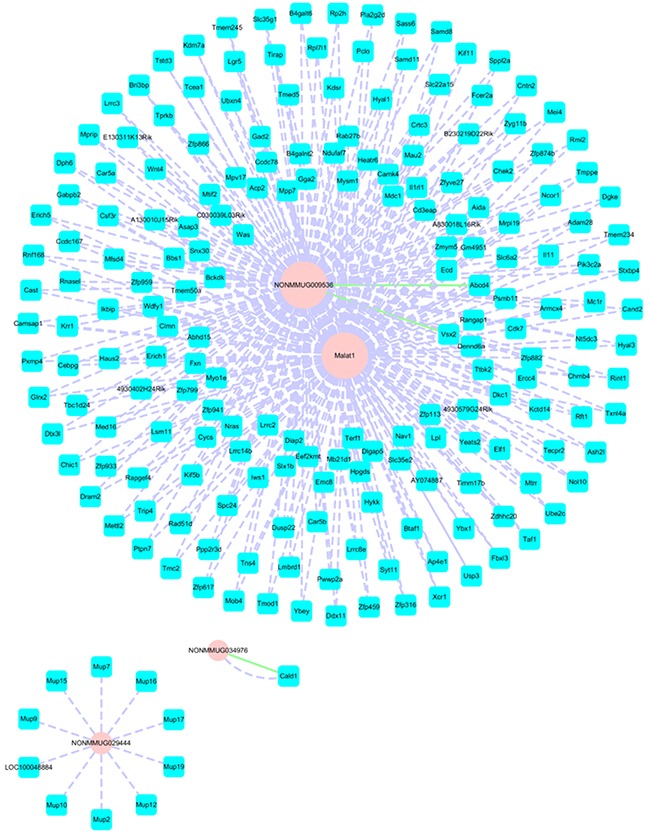
Coding-non-coding gene co-expression network (NONMMUG009526, Malat1, NONMMUG034976, and NONMMUG029444) Blue square and pink round nodes represent coding and non-coding genes; purple dashed and blue solid lines between the two nodes represent *trans* and *cis* targets, respectively. A larger point indicates that more targets are associated with the lncRNA.

The majority of the identified transcripts have unknown function. We selected four (NONMMUG009536, Malat1, NONMMUG029444, NONMMUG034976) lncRNAs for *cis* and *trans* target gene prediction. A coding-non-coding gene co-expression network was constructed from re-annotated Affymetrix Mouse Genome Array data (Figure 5).

### Quantitative real-time (qRT)-PCR validation

**Figure 6:**
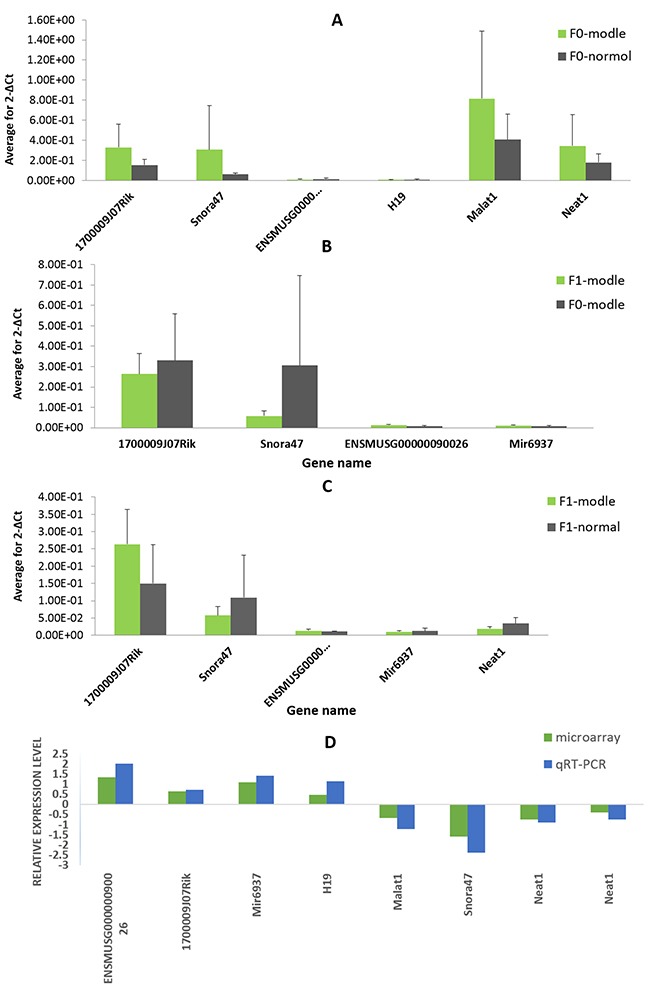
Validation of microarray data by quantitative reverse transcription-polymerase chain reaction (qRT-PCR) The relative expression levels of eight lncRNAs are shown comparing **(A)** F0-HFD/F0-normal; **(B)** F1-HFD/F0-HFD; and **(C)** F1-HFD/F1-normal. Data are presented as average 2^-ΔCt^, n = 10. **(D)** Comparison between qRT-PCR results and microarray data revealing a good correlation of the two methods. The heights of the columns represent the fold changes (log2 transformed) computed from the qPCR and microarray data.

Eight lncRNAs were chose for verification of the microarray results in three groups of samples by quantitative real-time PCR. qRT-PCR assay showed that the expression of lncRNA miR-6937, 1700009J07Rik, H19, and ENSMUSG00000090026 were upregulated, whereas nuclear-enriched abundant transcript (Neat)1, metastasis-associated lung adenocarcinoma transcription (Malat)1, and small nucleolar RNA (Snora)47 were downregulated. This result is consistent with the expression profiles from the microarray analysis (Figure 6). Hence, the qRT-PCR data verified the microarray results.

## DISCUSSION

Increasing evidence indicates that obesity induced by a high-fat diet can be inherited from the father, but the nature of the genetic vector inducing this newly acquired phenotype remains unclear. Identification of the genetic vector involved in obesity is an important step in addressing obesity. Our study showed that there was a significant difference in the expression of lncRNAs and mRNAs in spermatozoa of high-fat diet-induced obese mice and normal mice, and these differences were also present in their male offspring (F1). Interestingly, the opposite expression pattern was found in the F1-HFD and F0-HFD groups, where minimal differential expression of lncRNA and mRNA was observed. In other words, similar expression patterns of lncRNA and mRNA were retained in the F1-HFD/F0-HFD groups, suggesting that lncRNA acts as the hereditary vector inducing paternal inheritance of obesity.

Research has shown that RNA functions as a vector of epigenetic information [25]. However, their inheritance mechanism in the spermatozoa of obese mice remains unclear. LncRNAs are critical for spermatogenesis, and their dysregulation might lead to male infertility [22]. However, only a few lncRNAs have been studied in obese male mice. NEAT1 is expressed in human embryonic stem cells [26] and is involved in the regulation of spermatogenesis [27]. We found in our microarray analysis that the NEAT1 and Malat1 expression levels were downregulated in obese as compared to non-obese mice (NEAT1: F0-HFD/F0-NC = 0.18, F1-HFD/F1-NC = 0.4; Malat1: F0-HFD/F0-NC = 0.21, F1-HFD/F1-NC = 0.35), which were similar to previously reported values [27]. Decreased expression of NEAT1 is associated with reduced sperm quality and low fertility, and its expression is negatively regulated by Malat1 [27]. Together, these results suggest that high-fat diet-induced dysregulation of lncRNA expression promotes epigenetic changes that can remain phenotypically silent but are later transmitted to the progeny.

The GO analysis showed that lncRNAs that were differentially expressed between the F0-HFD and F0-NC groups were mostly highly enriched in functions associated with obesity-related reproductive pathogenesis, and the same differences were observed in their offspring. Specifically, “osteoclast differentiation” and “B cell receptor signalling pathway” have been linked to obesity, which is detrimental to bone formation and bone mineral density [28, 29]. Investigating the relationship between bone and fat metabolism can not only clarify the mechanisms common to obesity and osteoporosis, but also identify lncRNAs and other molecules associated with osteoclast differentiation that can serve as therapeutic targets in the treatment of these disorders. Furthermore, in the F1-HFD/F0-HFD groups, similarly expressed lncRNAs and mRNAs were mostly highly enriched in “positive regulation of somatostatin secretion”, “positive regulation of somatostatin secretion”, “steroid delta-isomerase activity”, and “integral component of membrane” GO terms. Thus, intergenerational inheritance of obesity may have occurred through mechanisms mediated by the similarly expressed mRNAs and lncRNAs.

It has been demonstrated in other species, that gonadotropin-releasing hormone (GnRH) signalling inputs could promote male mating-like behaviour [30]. The GnRH signalling pathway plays a key role in the central regulation of reproduction [31], and can regulate vital factors associated with fertility, such as leptin, insulin and ghrelin, which have been identified as the key mediators of “vital metabolic control” [32]. The Hippo signalling pathway represents a new molecular target for the regulation of organ-growth functional remodelling [33]. In the present research, we found that in the high-fat/normal groups, whether in the F0 or F1 generation, transcripts associated with GnRH and Hippo signalling pathway were upregulated, suggesting that the biological processes and regulatory mechanisms participating in the GnRH and Hippo signalling pathways may represent potential targets for the treatment of male infertility in obesity induced by a high-fat diet. Additionally, similarly expressed mRNAs in F1-HFD/F0-HFD were found to be closely linked with the G-protein coupled receptor signalling pathway, pancreatic polypeptide receptor activity, and lysine biosynthesis. The G-protein coupled receptor signalling pathway plays an important role in regulating mammalian reproduction and maintains oocyte meiosis by mitogen-activated protein kinase (MAPK) signalling [34]. Another study has demonstrated that a member of the pancreatic polypeptide family-Neuropeptide Y (NPY) could inhibit GnRH-1 neuronal activity through the G protein-coupled Y1 receptor (Y1R) in the early developmental stage [35]. This suggests that intergenerational obesity is inherited by offspring through these pathways associated with growth and development.

LncRNAs modulate gene expression in *cis* or in *trans* [36, 37]. In our study, two independent algorithms were used to predict the *cis* and *trans* target genes of differentially expressed lncRNAs in the sperm of obese mice. For example, the lncRNA showing the greatest difference in expression between F0-HFD and F0-NC in the NONMMUG009536 microarray was predicted to act both in *cis* (*ATP binding cassette subfamily D member* [*Abcd*] *4* and *visual system homeobox 2*) and in *trans* (*Rad51d* and *Wnt4*). Abcd4 is linked to vitamin B12 deficiency, which leads to a reduction in fat metabolism; the finding that *Abcd4* was downregulated in the F0-HFD group (F0-HFD/F0-NC = 0.46) is consistent with the fact that it could cause or aggravate obesity [38]. *Wnt4* may be involved in the pathogenesis of female infertility; spermatozoon-derived Wnt4 is transported to the fertilized egg and translated, and this process may be important in early embryonic development and provide a basis for the paternal gene effect [39]. Further studies are needed to confirm the functions of these differentially expressed lncRNAs and their regulation of *cis* and *trans* target genes in the sperm of obese mice offspring.

In conclusion, by analysing the expression of sperm lncRNA, we demonstrated that obesity was intergenerationally inherited by the offspring and similar expression patterns of lncRNAs in the F1 and F0 generation sperm may act as the hereditary vector mediating paternal heredity of obesity. Therefore, lncRNAs might serve as potential candidates for further comprehensive understanding and examination of obesity-induced male infertility as well as intergenerational inheritance of obesity.

## METHODS

### Ethics statement

The study protocol was approved by the Animal Care and Management Committee of the Beijing University of Chinese Medicine. All animal manipulations were according to the guidelines of the Animal Care Committee.

### Mice and diets

Male C57bl/6J mice (8 weeks old; Hua Fu Kang Company, Beijing, China) were used in this study. After 1 week of adaptive feeding, the mice were randomly divided into obesity model (HFD) and non-obese (NC) groups (n = 10). HFD groups were continuously fed an HFD (60% fat) for 10 weeks to induce obesity (body weight: 39–45 g; n = 10); after mating with fertile female mice, male offspring were reared for selection (n = 6). All mice other than half the F0 mice were maintained on SC feed.

### Sperm collection

Sperm was collected from F0-HFD (n = 6) and F0-NC (n = 6) mice and their male offspring (F1-HFD [n = 6] and F1-NC [n = 6], respectively). Mice were sacrificed by cervical dislocation, and sperm from the epididymis was transferred to a preheated human tubal fluid culture and centrifuged at 1,000 rpm for 5 min. Sperm capacitation was measured for 30 min, and the sperm supernatant was centrifuged again and collected.

### RNA extraction and purification

Total RNA was extracted and purified using miRNeasy Mini kit (cat. no. 1038703; Qiagen, Hilden, Germany) following the manufacturer's instructions. RNA integration was analysed with a Bioanalyzer 2100 (Agilent Technologies, Santa Clara, CA, USA).

### RNA amplification and labelling

Total RNA was amplified and labelled with the Low-Input Quick Amp WT Labeling kit (cat. no. 5190-2943; Agilent Technologies) following the manufacturer's instructions. Labelled cRNA was purified with the RNeasy Mini kit (cat. no. 74106).

### Microarray hybridisation

Each slide was hybridised with 1.65 μg Cy3-labelled cRNA using the Gene Expression Hybridization kit (cat. no. 5188-5242; Agilent Technologies) in a hybridisation oven (cat. no. G2545A; Agilent Technologies) according to the manufacturer's instructions. After 17 h of hybridisation, the slides were washed in staining dishes (cat. no. 121; Thermo Fisher Scientific, Waltham, MA, USA) using the Gene Expression Wash Buffer kit (cat. no. 5188-5327; Agilent Technologies) as instructed by the manufacturer.

### Data acquisition

Slides were scanned with the Microarray Scanner (cat. no. G2565CA; Agilent Technologies) using the following default settings: dye channel = green, scan resolution = 3 μm, PMT = 100%, and 20 bit. Data were extracted with Feature Extraction v.10.7 (Agilent Technologies). Raw data were normalised with the quantile algorithm of GeneSpring v.12.6.1 (Agilent Technologies). The microarray data presented in this article have been deposited in the National Center for Biotechnology Information Gene Expression Omnibus (GEO) and are accessible through GEO Series accession number GSE66783.

### Analysis of gene function

The differentially expressed genes were input into the Database for Annotation, Visualization, and Integrated Discovery (DAVID; http://david.abcc.ncifcrf.gov/) v.6.7, which used GO to identify the molecular functions represented in gene profiles and KEGG to analyse the potential functions of these genes in pathways [40, 41]. Lower *P* values represent more significant correlations; the recommended cut-off *P* value was 0.05.

### LncRNA target prediction

Differentially expressed lncRNAs were selected for target prediction as previously described [42]. We used two independent algorithms to identify target genes. The first searched for those acting in *cis*. Using University of California Santa Cruz (UCSC) gene annotations (http://genome.ucsc.edu/), lncRNAs and potential target genes were paired and visualised using the UCSC genome browser. Those transcribed within a 10-kbp window up- or downstream of the lncRNA were considered as *cis* target genes. The second algorithm is based on mRNA sequence complementarity and RNA duplex energy prediction, and evaluated the impact of lncRNA binding on complete mRNA molecules using BLAST for first-round screening. RNAplex was used to screen target genes in *trans* [43] with the RNAplex parameter set as −e^−20^.

### Coding-non-coding gene co-expression network

The majority of identified transcripts have unknown function. We constructed a coding-noncoding gene co-expression network that included the differentially expressed lncRNAs for *cis* and *trans* targeted coding genes from re-annotated Affymetrix Mouse Genome Array data.

### qRT-PCR

Total RNA was extracted from additional sperm samples of F0-HFD, F0-NC, F1-HFD, and F1-SC mice (n = 10 each) using RNAiso Plus (Takara Bio, Dalian, China). The RNA was quantified using a NanoDrop 1000 spectrophotometer (Thermo Fisher Scientific, Waltham, MA, USA). The ratio of absorbances at 260 and 280 nm (A260/A280 ≥ 1.8) was used to assess RNA purity. The RNA was then reverse transcribed using PrimeScript RT Master Mix (Takara Bio) according to the manufacturer's instructions. The eight differentially expressed lncRNAs were analysed by qRT-PCR using SYBR Premix Ex Taq II (Takara Bio) on the LightCycler 480 (Roche Diagnostics, Indianapolis, IN, USA) following the manufacturer's instructions, with glyceraldehyde 3-phosphate dehydrogenase (GAPDH) used as an internal control. Amplified genes and primers used in this study are shown in Table 3. The relative expression level of each lncRNA was calculated using double-standard curves. Experiments were performed in triplicate.

**Table 3:** PCR primers used for expression studies

Gene		Primer Sequence (5’ to 3’)
Primers for lncRNAs		
H19	Forward	GTGTGGCCGTGTGCTTGAG
	Reverse	GTAGGGCATGTTGAACACTTTATGA
1700009J07Rik	Forward	AGAAGCCAGCCACCACTAAG
	Reverse	GACAGGACCAGCTTGCTTTC
Neat1	Forward	GGGAAAGCTGTTGGGTTGTA
	Reverse	CGGCAGAATTTGTGGCTAAC
Snora47	Forward	CCGTGCTGCCTTCCATTG
	Reverse	CCACGGTGATAGAGAGGACATTC
Malat1	Forward	TGCTGCATTAAGCCTGGAGT
	Reverse	GAAACATTGGCACACTGCAC
Neat1	Forward	TGGGGAAATGTGAAGAAAGC
	Reverse	TTGCTGTAAAGGGGAGGAAA
ENSMUSG00000090026	Forward	TTGACTACCTTTGGCACCG
	Reverse	AAGCAAGCTCTGGTCTCTTCTG
Mir6937	Forward	GTAAGGGCTGGGTCTGTGTG
	Reverse	CTAGTGCAAGGGGGAACCT
Primers for internal control		
Gapdh(mouse)	Forward	CACAATTTCCATCCCAGACC
	Reverse	GTGGGTGCAGCGAACTTTAT

### Statistical analysis

Data are expressed as mean ± standard deviation. The threshold value used to screen differentially expressed lncRNAs and mRNAs was fold change > 2.0 (*P* < 0.05). Differences between means were evaluated with the Student's *t* test using SPSS v.22.0 (SPSS Inc., Chicago, IL, USA). P < 0.05 was considered statistically significant.

## SUPPLEMENTARY MATERIALS AND TABLES






