# Correction: Long non-coding RNAs could act as vectors for paternal heredity of high fat diet-induced obesity

**DOI:** 10.18632/oncotarget.26635

**Published:** 2019-01-22

**Authors:** Tian An, Teng Zhang, Fei Teng, Jia-Cheng Zuo, Yan-Yun Pan, Yu-Fei Liu, Jia-Nan Miao, Yu-Jie Gu, Na Yu, Dan-Dan Zhao, Fang-Fang Mo, Si-Hua Gao, Guangjian Jiang

**Affiliations:** ^1^ Diabetes Research Center, Beijing University of Chinese Medicine, Beijing, China; ^2^ State Key Laboratory of Stem Cell and Reproductive Biology, Institute of Zoology, Chinese Academy of Sciences, Beijing, China; ^3^ The Third Affiliated Hospital, Beijing University of Chinese Medicine, Beijing, China

**This article has been corrected:** The correct GEO number information is given below:

**Data acquisition**

Slides were scanned with the Microarray Scanner (cat. no. G2565CA; Agilent Technologies) using the following default settings: dye channel = green, scan resolution = 3 μm, PMT = 100%, and 20 bit. Data were extracted with Feature Extraction v.10.7 (Agilent Technologies). Raw data were normalised with the quantile algorithm of GeneSpring v.12.6.1 (Agilent Technologies). The microarray data presented in this article have been deposited in the National Center for Biotechnology Information Gene Expression Omnibus (GEO) and are accessible through GEO Series accession number GSE118039.

Original article: Oncotarget. 2017; 8:47876-47889. https://doi.org/10.18632/oncotarget.18138

