# Supplementary material for: Long non-coding RNAs could act as vectors for paternal heredity of high fat diet-induced obesity
Source: Oncotarget. 2017 May 24;8(29):47876–89. doi: 10.18632/oncotarget.18138 (PMC5564612; doi:10.18632/oncotarget.18138)
Supplement: Supplementary file 1 [file oncotarget-08-47876-s001.pdf]

Long noncoding RNAs could act as vectors for paternal heredity of high fat diet-induced obesity

SUPPLEMENTARY MATERIALS

Supplementary Table 1: Gene ontology (GO) analysis of differentially expressed mRNAs.

See Supplementary File 1

Supplementary Table 2: Pathways analysis of differentially expressed mRNAs.

See Supplementary File 2
